# Disulfiram alleviates pristane-induced lupus via inhibiting GSDMD-mediated pyroptosis

**DOI:** 10.1038/s41420-022-01167-2

**Published:** 2022-09-03

**Authors:** Lili Zhuang, Xiaoqing Luo, Shufan Wu, Zhangmei Lin, Yanan Zhang, Zeqing Zhai, Fangyuan Yang, Yehao Li, Jian Zhuang, Guihu Luo, Wenchao Xu, Yi He, Erwei Sun

**Affiliations:** 1 Department of Rheumatology and Immunology, The Third Affiliated Hospital, Southern Medical University, Guangzhou, China; 2 Department of Rheumatology and Immunology, Shunde Hospital, Southern Medical University, Foshan, China

**Keywords:** Inflammasome, Lupus nephritis

## Abstract

Activation of multiple inflammasomes in monocytes/macrophages is associated with the pathogenesis of systemic lupus erythematosus (SLE). Gasdermin D (GSDMD)-mediated pyroptosis, a common consequence of multiple activated inflammasomes, is a programmed cell death with strong inflammatory responses. This suggested that targeting monocyte/macrophage pyroptosis might provide an opportunity to cure SLE. Here, we aimed to investigate the effect of disulfiram (DSF), a small molecule inhibitor of pyroptosis, and its potential therapeutic mechanism for SLE. The mRNA expression of GSDMD and IL-1β were significantly increased in peripheral blood mononuclear cells (PBMCs) from SLE patients. Importantly, we found serum from SLE patients rather than healthy controls induced GSDMD-mediated pyroptosis in THP-1 cells, as evidenced by enhanced LDH release, increased number of PI-positive cells, and high expression of full-length GSDMD and N-terminal GSDMD. Interestingly, treatment with DSF obviously inhibited pyroptosis of THP-1 cells induced by serum from SLE patients. Of note, DSF administration reduced proteinuria, serum anti-dsDNA level, and renal immune complex. It also attenuated renal damage in PIL mice. Further research found that the high level of serum IL-β and GSDMD-mediated pyroptosis of glomerular macrophages in PIL mice were rescued with DSF treatment. These data implied that GSDMD-mediated monocytes/macrophages pyroptosis played an important role in the pathogenesis of SLE and DSF might be a potential alternative therapeutic agent for SLE.

## Introduction

Systemic lupus erythematosus (SLE) is a systemic autoimmune disease characterized by the production of autoantibodies, the deposition of immune complexes, and the infiltration of immune cells [1, 2]. SLE has diverse clinical manifestations affecting almost any organ system, particularly the kidneys, the skin, and the nervous system [3, 4]. However, the etiology of SLE remains elusive up to now. Recently, emerging data highlighted the key role of innate immune system in the pathogenesis of SLE [5, 6]. Monocytes/macrophages, the frontline innate immune cells, were closely correlated with disease activity and poor outcomes in SLE patients [7–10].

Pyroptosis, a type of programmed necrosis, is characterized by large bubbles blowing from the plasma membrane, cell lysis, and release of pro-inflammatory intracellular contents [11]. It was first found in macrophages infected with Shigella flexneri [12] and thought to be caspase-1-mediated monocyte/macrophage death for a long time [11]. Recent studies identified that pyroptosis was directly caused by gasdermin family proteins [13] and not cell-type specific [14–17]. Gasdermin D (GSDMD), a member of gasdermin family, is the substrate of caspase-1 and caspase-4/5/11. The activation of caspase-1 by multiple inflammasomes including the NLRP3, NLRC4, NLRP1, AIM2, or Pyrin cleaves GSDMD into an N-terminal GSDMD fragment (GSDMD-NT). The same cleavage of GSDMD was also observed with caspase-4/5/11 upon recognition of cytosolic lipopolysaccharide (LPS), the major component of the gram-negative bacterial cell wall [11]. GSDMD-NT further forms membrane pores, resulting in cell rupture and release of the intracellular contents, such as IL-1β and IL-18, to promote the inflammatory response [13, 18]. Numerous studies have shown that pyroptosis might be closely related to occurrence of many diseases [19–21]. For example, serum of rheumatoid arthritis (RA) patients could induce GSDMD-dependent pyroptosis in monocytes, which was promoted by PTX3 and C1q.

Recent advances strongly hint that GSDMD-mediated monocyte/macrophage pyroptosis might be an effective drug target for treating SLE. For a long time, the Lupus erythematosus (LE) cell has been an important biomarker for systemic lupus erythematosus and a standard for diagnosis. Past studies have indicated that intact nuclei were released during pyroptosis, a process that is tightly linked to LE cells formation [22]. In both male and female SLE patients, NLRP3 inflammasome was highly activated in macrophages [23]. Anti-dsDNA autoantibody, a marker SLE, could activate NLRP3 inflammasome in monocytes/macrophages and further amplify inflammatory responses [24]. It was also found that the expression of AIM2 in peripheral blood mononuclear cells (PBMCs) of lupus patients significantly increased, and was positively correlated with disease activity. Also, AIM2 expression was increased in kidney macrophages of lupus mice, and knockdown of AIM2 significantly ameliorated tissue damage by inhibiting macrophages activation [25]. Furthermore, the protein expression of GSDMD was remarkably elevated in kidney specimens of SLE patients and MRL/lpr mice. Intriguingly, combination therapy suppressed disease progression by attenuating GSDMD-mediated pyroptosis [26]. Taken together, in monocytes/macrophages of SLE patients, abnormal activation of various inflammasomes might trigger the formation of pro-inflammatory GSDMD-NT to mediate pyroptosis, which ultimately contributed to the development of SLE.

Disulfiram (DSF) is widely used to treat alcohol addiction by inhibiting aldehyde dehydrogenase for decades [27, 28]. Recently, DSF was reported to be an inhibitor of GSDMD-mediated pyroptosis, mainly by suppressing inflammatory caspase activation, the expression of GSDMD, and GSDMD pore formation [29–32]. A subsequent study showed that DSF protected experimental autoimmune encephalomyelitis (EAE) mice by inhibiting GSDMD protein expression [33]. In this study, we aimed to determine whether GSDMD-mediated monocyte/macrophage pyroptosis promoted the development of SLE and DSF had a therapeutic effect on pristane-induced lupus (PIL) mice model.

## Results

### High mRNA expression of GSDMD and IL-1β in PBMCs from SLE patients

**Fig. 1:**
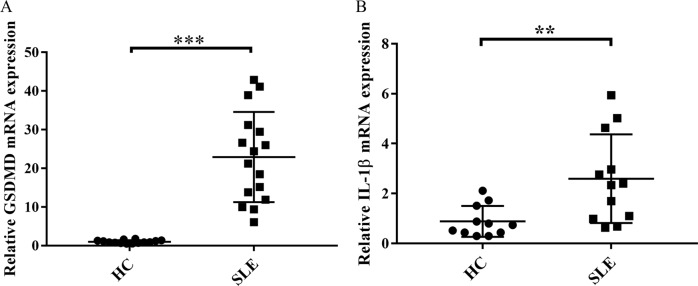
mRNA expression of GSDMD and IL-1β was increased in PBMCs from SLE patients. **A** GSDMD mRNA expression in PBMCs from SLE patients (*n* = 16) and healthy controls (HC, *n* = 14). **B** mRNA expression of IL-1β in PBMCs from SLE patients (*n* = 12) and HC (*n* = 11). mRNA determination was performed by real-time PCR. Values were shown as mean ± SD of three independent experiments. **p < 0.01, ***p < 0.001. HC healthy controls, SLE Systemic lupus erythematosus.

Previous studies have shown that activated inflammasomes are closely related to the severity of the disease in SLE patients [25, 34–36], suggesting that GSDMD-mediated pyroptosis might be involved in SLE. We first determined the expression of GSDMD gene in PBMCs from SLE patients and healthy controls by real-time PCR analysis. As shown in Fig. 1A, the expression levels of GSDMD mRNA in PBMCs from SLE patients were significantly higher than that from healthy controls. As the process of pyroptosis is accompanied by increased expression of IL-1β [11, 37], we next confirmed IL-1β gene expression in PBMCs from SLE patients and healthy controls. Indeed, we found that PBMCs from SLE patients expressed higher levels of IL-1β mRNA than PBMCs from healthy controls (Fig. 1B). These results suggested that SLE patients might express high mRNA of GSDMD and IL-1β in PBMCs.

### DSF significantly inhibited GSDMD-mediated pyroptosis of THP-1 cells induced by serum from SLE patients

PMBCs contain many different cells, including monocytes. Previous studies have found that abnormal activation of various inflammasomes existed in monocytes of SLE patients. Therefore, we believed that monocytes in the PBMCs of SLE patients might undergo GSDMD-mediated pyroptosis. In the following experiment, we used serum from SLE patients, with and without mixing DSF, to stimulate human monocyte line THP-1 and then observed whether the cells underwent pyroptosis.

**Fig. 2:**
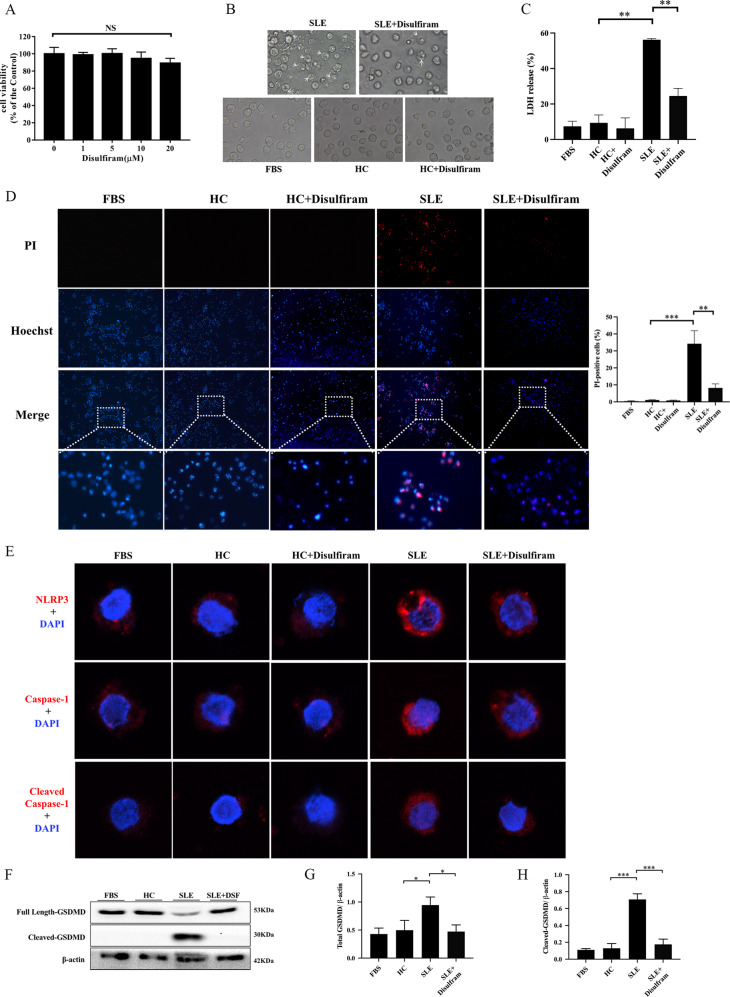
GSDMD-mediated pyroptosis of THP-1 cells induced by serum from SLE patients was suppressed by DSF. **A** Effect of DSF on the cell viability of THP-1 cells. Cells were treated with DSF (0, 1, 5, 10, 20 μM) for 48 h and then cell viability measured by CCK-8 assay. **B** The morphological features of THP-1 cells treated with serums from healthy controls or SLE patients, with or without mixing 10 μM disulfiram. **C** Lactate dehydrogenase (LDH) release from THP-1 cells treated as indicated. **D** Hoechst33342/Propidium Iodide (PI) double staining in THP-1 cells after different treatments. **E** Representative immunofluorescence images showing the expression of NLRP3, caspase-1, and cleaved caspase-1 in THP-1 cells treated as indicated. **F** The expression of full length and cleaved GSDMD in THP-1 cells. The cells were incubated in different mediums and analyzed by western blot analysis. β-actin was used as a protein loading control. **G**, **H** The expression level of total GSDMD and cleaved-GSDMD relative to β-actin were quantified. Total GSDMD = full length-GSDMD + cleaved-GSDMD. Significant differences were calculated using one-way ANOVA. Values were shown as mean ± SD. *p < 0.05, **p < 0.01, ***p < 0.001. Each experiment was repeated three times. FBS fetal bovine serum, HC Healthy control, NS not significant, PI propidium iodide, LDH Lactate dehydrogenase.

We first analyzed the cytotoxicity of DSF on THP-1 cells using CCK-8 assay. The results showed that DSF (1, 5, 10, 20 μM) did not have a significant effect on cell viability (Fig. 2A). Based on this and previous results [30], we used 10 μM for subsequent vitro experiments. Then the THP-1 cells were stimulated with serum from healthy controls or SLE patients, with and without mixing 10 μM DSF.

When THP-1 cells were treated with serum from SLE patients, they became swelling and blew out large bubbles from the plasma membranes (Fig. 2B), the typical cell morphological character of pyroptosis. Also, LDH release in supernatant and the number of PI-positive cells increased in THP-1 cells treated with serum from SLE patients (Fig. 2C, D), indicating that THP-1 cells died due to the loss of cell membrane integrity.

Next, to further determine whether cell death induced by serum from SLE patients was mediated by GSDMD-dependent pyroptosis, we tested the expression of NLRP3, caspase-1, and cleaved caspase-1 by immunofluorescent staining, and GSDMD by western blot. As shown in Fig. 2E–H, the expression of NLRP3, caspase-1, and cleaved caspase-1, total GSDMD, and cleaved GSDMD increased after treatment with serum from SLE patients. Strikingly, DSF significantly reduced bubbles, LDH release, the number of PI-positive cells, and the expression of NLRP3, caspase-1, cleaved caspase-1, total GSDMD, and cleaved GSDMD (Fig. 2B–H). Collectively, these results implied that GSDMD-dependent monocyte pyroptosis might occur in peripheral blood of SLE patients, and be inhibited by DSF.

### DSF ameliorated the disease activity in PIL mice

**Fig. 3:**
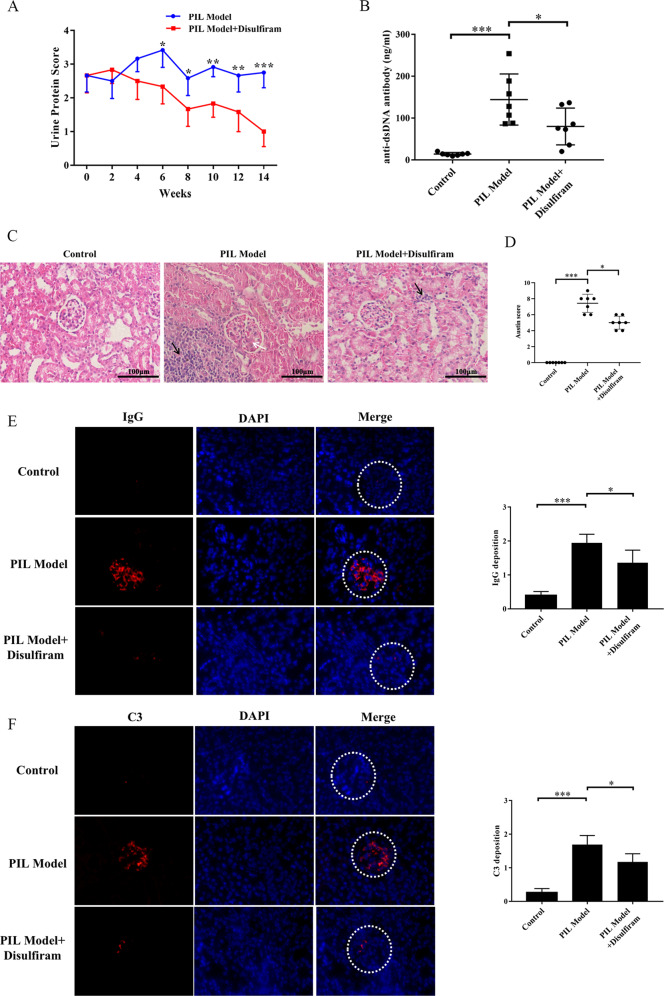
DSF ameliorated lupus-associated manifestations in PIL mice. PIL mice were treated with a dose of 50 mg/kg DSF or equivalent sterile saline for 14 weeks. **A** Proteinuria of mice in each group was assessed using Albustix test every 2 weeks. **B** The level of serum anti-dsDNA antibodies was examined by ELISA. **C** Representative photographs of the kidneys from each group by H&E staining. Inflammatory cell infiltration was with black arrows, and mesangial cell proliferation was with white arrow. **D** Austin scores of kidneys from each group. **E**, **F** Representative images in glomeruli of kidneys from each group stained for the deposition of IgG and C3. *n* = 8 animals for each group. *p < 0.05, **p < 0.01, ***p < 0.001. PIL pristane-induced lupus, IgG immunoglobulin G, C3 Complement 3.

To determine the therapeutic effect of DSF on PIL mice, BALB/c mice were injected intraperitoneally with pristane and then treated with a dose of 50 mg/kg DSF or equivalent sterile saline. As shown in Fig. 3A, B, DSF significantly reduced proteinuria and serum level of anti-dsDNA antibodies in PIL mice. In addition, DSF mitigated inflammatory cell infiltration, mesangial cell proliferation, and structural disorder of renal tubules in the kidney of PIL mice (Fig. 3C, D). Similarly, the deposition of IgG and C3 notably decreased in the kidney of DSF-treated PIL mice (Fig. 3E, F). Taken together, these findings demonstrated that DSF could rescue lupus-associated renal impairment in PIL mice.

### DSF inhibited glomerular macrophage pyroptosis in PIL mice

**Fig. 4:**
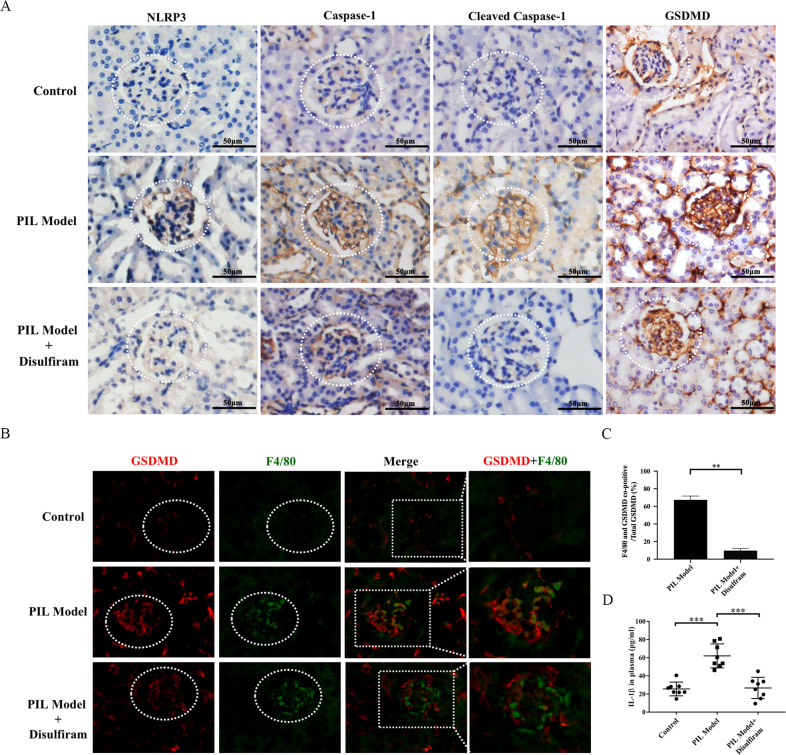
DSF reduced the level of serum IL-1β and inhibited glomerular macrophage pyroptosis in PIL mice. **A** Representative immunohistochemical staining images of NLRP3, caspase-1, cleaved caspase-1 and GSDMD in the kidneys. **B** Double immunofluorescence staining of glomerular macrophages (green) and GSDMD (red) in each group. F4/80 represented macrophages. **C** GSDMD in monocytes/macrophages as a proportion of total GSDMD was calculated. **D** The level of serum IL-1β in each group was detected by ELISA. *n* = 8 animals for each group. Each experiment was repeated three times. Significant differences were calculated using one-way ANOVA. Values were shown as mean ± SD. **p < 0.01, ***p < 0.001. PIL pristane-induced lupus. DAPI 4′, 6-diamidino-2-phenylindole.

As the process of pyroptosis amplifies inflammatory response by releasing IL-1β and other Inflammatory factors [11, 37], we detected the level of serum IL-1β. We found that the serum levels of IL-1β increased in PIL mice, while DSF remarkably inhibited the release of IL-1β (Fig. 4D). The kidney is a vulnerable organ for SLE patients, and approximately 60% of SLE patients are suffering from lupus nephritis (LN). To demonstrate the involvement of GSDMD-mediated pyroptosis in renal lesions of PIL mice, the expression of NLRP3, cleaved caspase-1, caspase-1, and GSDMD were examined. As expected, a significant increase in the expression of NLRP3, cleaved caspase-1, caspase-1, and GSDMD in the glomerulus could be detected in the kidneys of PIL mice, which was suppressed by DSF (Fig. 4A). To further confirm whether the macrophages underwent GSDMD-mediated pyroptosis in the glomerulus, an immunofluorescence co-localization assay was performed. In the PIL mice, the expression of GSDMD in glomerular macrophages increased significantly (Fig. 4B). Of note, DSF weakened the intensity of immunofluorescent staining of GSDMD in the glomerular macrophages of lupus mice (Fig. 4B). Furthermore, we calculated the proportion of GSDMD in macrophages to the total amount of GSDMD in whole glomerular region and found that GSDMD is mainly expressed in monocytes/macrophages (Fig. 4C). These results indicated DSF might inhibit glomerular macrophage pyroptosis in PIL mice.

## Discussion

SLE is a multifactorial systemic autoimmune disease, characterized by production of abundant autoantibodies [3]. Increasing evidence has demonstrated that dysregulated cell death and defective removal of dead cells underlie the onset of SLE [38–40]. In SLE patients, defective removal of dead cells contributes to the exposure of autoantigens and the release of damage-associated molecular patterns (DAMPs), thereby amplifying inflammation and immune responses [4]. Multiple inflammatory deaths have been found to be important in SLE pathogenesis and progression, such as NETosis, necroptosis, and secondary necrosis after apoptosis. NETosis, a specialized form of cell death in neutrophils, might lead to tissue damage in patients with SLE [41, 42]. Some animal studies proved that inhibition of NETosis could protect against lupus-related damage to kidneys, and skin in lupus-prone mouse models [43, 44]. Necroptosis is a form of programmed necrotic cell death mediated by the receptor-interacting protein kinase (RIPK) 1/RIPK3/mixed lineage kinase domain-like protein (MLKL) pathway [45]. Previous studies found that RIP3-dependent necroptosis was activated in SLE patients and inhibition of RIP3 kinase could inhibit the development of LN in MRL/lpr mice [46, 47].

Pyroptosis is another programmed cell death that could cause cell lysis and amplify inflammatory responses. Emerging studies have shown that pyroptosis might participate in the progression of SLE [22–24, 26]. Our study provided new evidence for the involvement of GSDMD-mediated monocytes/macrophages pyroptosis in SLE. Another finding is that DSF might ameliorate disease severity of PIL mice by suppressing monocytes/macrophages pyroptosis.

Monocytes/macrophages are innate immune cells with diverse biological functions including antimicrobial effect, antigen processing and presentation, clearance of apoptotic cells, wound healing, and promotion of inflammatory responses [48–51]. Emerging data have revealed that monocytes/macrophages were associated with disease activity and poor prognosis in SLE [7–9]. Further studies found that multiple inflammasomes, such as NLRP3 or AIM2, were activated in monocytes/macrophages of SLE patients [23–25]. More importantly, blocking or regulating macrophage inflammasome activity could attenuate disease progression in murine lupus [25, 52], suggesting that regulating the common downstream signaling of inflammasome activation might be the key to treatment of SLE.

Activation of different inflammasomes could collectively trigger pro-caspase-1 to form caspase-1, resulting in maturation of inflammatory factors such as IL-1β [53, 54]. Also, activated caspase-1 cleaves GSDMD to form GSDMD-NT, mediating the process of a programmed death, pyroptosis [13]. According to the cell death recognition model for the immune system, apoptotic cells induce immune tolerance and necrotic cells promote immune responses [55–58]. Pyroptosis as a form of programmed necrosis can elicit a robust inflammatory reaction and lead to target organs injury. The pore-forming protein GSDMD as the final pyroptosis executioner downstream of inflammasome activation controls pro-inflammatory cytokine release, such as IL-1β. IL-1β is considered to be an important pro-inflammatory cytokine that can drive inflammation, cause tissue damage and fibrosis, and amplify the effects of other cytokines. In the present study, we first found that the mRNA expression of GSDMD and IL-1β were significantly increased in PBMCs from SLE patients. Importantly, serum isolated from SLE patients could promote the expression and activation of GSDMD to induce pyroptosis in THP-1 cells. In addition, we also found serum level of IL-1β and GSDMD-mediated pyroptosis of glomerular macrophages were elevated in PIL mice. From the results of immunofluorescence staining, we found that other cells in the glomerulus of PIL mice also marginally expressed GSDMD, indicating these cells in the glomerulus might undergo pyroptosis. However, we quantified the ratio of GSDMD expression in macrophages and found that macrophages were the main cells expressing GSDMD in the glomerulus of PIL mice. These data indicate that GSDMD-mediated pyroptosis of macrophages might have a pivotal role in the initiation of SLE.

DSF, an inexpensive and safe drug, has been widely used for the treatment of alcohol addiction by inhibiting acetaldehyde dehydrogenase in the clinic. Over the past several decades, DSF was demonstrated to have strong anti-cancer activity both in vivo and vitro. Further research found that DSF exerted a tumor suppressor effect by regulating different pathways in cancer cells, including inhibiting the proteasome and changing the MAPK or MMP pathway. Recently, it has been discovered to prevent GSDMD-mediated pyroptosis by affecting multiple steps including inflammatory caspase activation, the expression of GSDMD, and GSDMD pore formation. In this study, we explored the therapeutic potential of DSF against SLE. As expected, DSF treatment could alleviate lupus-like features in PIL mice, as evidenced by reduction of serum anti-dsDNA antibodies level and the deposition of renal immune complex, and improvement of the pathological damage to kidneys. To reveal the mechanism of DSF in the treatment of SLE, we investigated whether DSF could inhibit monocytes/macrophages pyroptosis. As a result, DSF effectively inhibited GSDMD-mediated pyroptosis of THP-1 cells induced by serum from SLE patients. In vivo experiments, we further found that DSF treatment reduced the release of serum IL-1β and suppressed glomerular macrophage pyroptosis in PIL mice. All the above investigations demonstrated that DSF might protect PIL mice against lupus-like symptoms via inhibiting monocytes/macrophages pyroptosis and also provided further support for the role of monocytes/macrophages pyroptosis in SLE. Nowadays, many mechanisms have been found that might contribute to SLE development, so DSF might protect PIL mice from lupus-like symptoms via multiple mechanisms, and abrogating monocytes/macrophages pyroptosis can be one of them.

Recently, several studies have revealed divergent results on the role of GSDMD in SLE. On the one hand, consistent with our findings, expression and activation of GSDMD were increased in kidney specimens of SLE patients and lupus mice, which could be inhibited by combination therapy of different immunosuppressive agents [26]. On the other hand, in a TLR7-induced model of SLE, GSDMD^−/−^ mice developed more severe kidney damage and produced more autoantibodies [59]. The possible reason was that in the absence of GSDMD, the NLRP3 inflammasome might cooperate with caspase-3/8 to cause GSDME-induced pyroptosis [60, 61]. In our experiment, DSF could simultaneously reduce the expressions of NLRP3 inflammasome and GSDMD, so it might avoid the occurrence of GSDME-mediated pyroptosis caused by inflammasome activation.

In conclusion, Our study reveals that GSDMD-mediated monocytes/macrophages pyroptosis represents a therapeutically targetable mechanism in SLE and DSF might have protective effects against SLE. These findings open up new perspectives for understanding the molecular mechanisms and identifying the potential therapeutic intervention of SLE.

## Materials and methods

### Reagents

Antibodies included mouse GSDMD (Affinity Biologicals, catalog AF4012, Ancaster, Canada), human GSDMD and cleaved GSDMD (Abcam, catalog ab215203 and ab210070, Cambridge, MA, USA), β-actin (ProteinTech Group, catalog 66009, Chicago, IL), horseradish peroxidase (HRP)-conjugated secondary antibodies (Jackson ImmunoResearch, catalog 111-035-003 and 115-035-003, West Grove, PA), NLRP3 (Affinity Biologicals, catalog DF7438, Ancaster, Canada), caspase-1 (Santa Cruz Biotechnology, catalog sc392736, Dallas, TX, USA), cleaved caspase-1 (Affinity Biologicals, catalog AF4022, Ancaster, Canada), F4/80 (Santa Cruz Biotechnology, catalog sc377009, Dallas, TX, USA), IgG conjugated to Alexa Fluor 555 (Abcam, catalog ab150114, Cambridge, MA, USA) and rabbit anti-mouse C3 antibody (Abcam, catalog ab97462 Cambridge, MA, USA).

Pristane, Hoechst33342, and propidium iodide(PI) were purchased from sigma-aldrich (St. Louis, MO, USA). PrimeScript RT Reagent Kit and SYBR Green PCR Kit were obtained from TaKaRa (Tokyo, Japan). Other reagents included 1% Penicillin-streptomycin (Gibco Laboratories, Grand Island, NY, USA), lymphocyte separation medium (TBD Sciences, Tianjin, China), TRIzol Reagent (Invitrogen, Carlsbad, CA, USA), RPMI-1640 medium (South Logan, UT, USA), Cell Counting Kit (CCK8, Dojindo, Kumamoto, Japan), the CytoTox 96 Non-Radioactive Cytotoxicity Assay (Promega, Madison, WI, USA), hematoxylin and eosin (H&E, Boster Biological Technology co. ltd, Wuhan, China), DSF (APExBio, Houston, TX, USA), RIPA buffer (Beyotime, Shanghai, China), BCA protein assay kit (Thermo Fisher Scientific, Waltham, MA, USA), DAB working solution (Noble Ryder Technology Co.Ltd, Beijing, China), 4′, 6-diamidino-2-phenylindole (Thermo Fisher Scientific, Waltham, MA, USA) and enzyme-linked immunosorbent assay (ELISA) kits for IL-1β (NeoBioscience Technology Company, Shenzhen, China) and anti-dsDNA antibodies (Cusabio Life Science Inc., Wuhan, China).

### Mice

Female BALB/c mice, at 6-8 weeks of age, were purchased from Guangdong Medical Laboratory Animal Center (GDMLAC, Guangdong, China) and housed under a pathogen-free condition with a 12-h light and dark cycle in the laboratory animal center of Southern Medical University. All procedures for animals experiment were approved by the Southern Medical University Experimental Animal Ethics Committee (No. L2017032).

### PIL mouse model

BALB/c mice were injected intraperitoneally (i.p.) with 0.5 ml pristane and proteinuria was assessed using Albustix test every 2 weeks. The following scale was used for semi-quantitative evaluation: 0 score = absent, 1 score = 300–1000 mg/L, 2 score = 1000–3000 mg/L, 3 score = 3000–20,000mg/L, and 4 scoreå 20,000mg/L. When all BALB/c mice had obvious proteinuria, they were randomly divided into two groups: the PIL group (*n* = 8), and the PIL + DSF group (*n* = 8). The mice in the PIL + DSF group were intraperitoneally treated with a dose of 50 mg/kg DSF for another 14 weeks. In the PIL group, the mice were injected with equivalent sterile saline daily. Eight normal female BALB/c mice received the same sterile saline intraperitoneally as control.

### Isolation of human peripheral blood mononuclear cells (PBMCs)

Human PBMCs were isolated from peripheral blood of SLE patients and healthy controls using a lymphocyte separation medium according to the manufacturer’s instruction. SLE patients were from the Third Affiliated Hospital of Southern Medical University and fulfilled the classification criteria of American College of Rheumatology (ACR) for SLE. Healthy controls matched the SLE patients, including age and gender. We obtained informed consent from all participants.

### Real-time PCR analysis

Total RNA was extracted from PBMCs using TRIzol Reagent and reversely transcribed into cDNA using the PrimeScript RT Reagent Kit according to the corresponding instructions. The expression of the genes encoding GSDMD and IL-1β was quantified by real-time PCR using the SYBR Green PCR Kit following the manufacturer’s protocol and GAPDH used as a loading control. The PCR was performed under the following standard thermal conditions: 30 s at 95 °C, 40 cycles at 95 °C for 30 s, and 34 s at 60 °C. All samples were replicated in parallel three times and PCR reactions performed in triplicate. The level of gene encoding expression was calculated using SDS software (Applied Biosystems). The primer sequences are listed in Table 1.

**Table 1:** The sequences of PCR primers.

Gene	Sequences
Human GSDMD	Forward: 5′-AGCCCTACTGCCTGGTGGTTAG-3′
	Reverse: 5′-CCTGCGATCTTTGCCTGTCCTG-3′
Human IL-1β	Forward: 5′-GCGGCATCCAGCTACGAATCT-3′
	Reverse: 5′-CGGAGCGTGCAGTTCAGTGAT-3′
Human GAPDH	Forward: 5′-CAAGGCTGTGGGCAAGGTCAT-3′
	Reverse: 5′-AGTGGGTGTCGCTGTTGAAGTC-3′

### Collection of serum samples and cell culture

Peripheral venous blood (5 ml) was collected in a tube filled with procoagulants from each SLE patient or healthy control. After the blood samples were centrifuged at 500 *g*/r for 10 min, the supernatant was collected and centrifuged at 1500 *g*/min for 10 min to get the upper serum.

The THP-1 cells, human acute monocytic leukemia cell line, were provided by American Type Culture Collection (ATCC, Manassas, VA, USA) and cultured in RPMI-1640 medium supplemented with 10% fetal bovine serum (FBS) and 1% Penicillin-streptomycin at 37 °C under 5% CO_2_. The THP-1 cells were treated with 10% serum from randomly mixed five SLE patient samples or five healthy control samples with or without 10 μM DSF for 48 h, and the cell morphology was observed with an inverted microscope.

### Cell viability assay

The effect of DSF on THP-1 cells viability was measured using a CCK8 following the manufacturer’s instructions. THP-1 cells were incubated at different concentrations of DSF (0 μM, 1 μM, 5 μM, 10 μM, 20 μM) for 48 h and then 10 μl CCK-8 reagent was added to cells followed by additional 1–4 h incubation. Finally, the absorbance at 450 nm was measured.

### Hoechst33342 and PI double staining

Cell cytotoxicity was determined by Hoechst33342 and PI double staining. THP-1 cells were cultured in 10% serum from SLE patients and healthy controls, with or without 10 μM DSF for 48 h, and washed twice with phosphate buffered saline (PBS). After that, cells were stained with Hoechst33342 and PI for 15 minutes in the dark and then observed and photographed under an inverted fluorescence microscope (Carl Zeiss, Jena, Germany).

### Lactate dehydrogenase (LDH) assay

Cell death after different treatments was assessed by quantifying LDH release from supernatants using the CytoTox 96 Non-Radioactive Cytotoxicity Assay according to the manufacturer’s instructions.

### Analysis of serum samples

The ELISA kits were used to measure the serum levels of IL-1β and anti-dsDNA antibodies following the manufacturer’s protocols.

### Assessment of renal injury

All mice were anesthetized with a lethal dose of pentobarbital and then their kidney tissues collected. The left kidney tissues were fixed in 10% formalin, then embedded in paraffin and sectioned at 4μm- thickness with a section cutter. Then the sections were stained with H&E according to the manufacturer’s instructions. Renal injury was scored by pathologists who were blinded to the experimental information using the Austin score as previously described [62].

Some right kidney tissues were made into 4 μm thick frozen sections for evaluation of glomerular IgG and complement C3 deposition. The frozen sections were blocked with 5% goat serum for 1 h and stained with goat anti-mouse IgG conjugated to Alexa Fluor 555 and rabbit anti-mouse C3 antibody in combination with anti-rabbit IgG conjugated to tetramethylrhodamine isothiocyanate (TRITC). The different intensity of IgG/C3 deposition was evaluated as 0-3 scores, where 0 indicates no deposition and 3 represents strong deposition, as previously described methods [63, 64].

### Western blot analysis

Protein levels of full-length GSDMD and cleaved GSDMD were determined by western blot. Firstly, cells were lysed in RIPA buffer containing protease inhibitor phenylmethanesulfonyl fluoride (PMSF) for half an hour. Proteins were quantified using the BCA protein assay kit. Then proteins were separated on 12% SDS polyacrylamide gel and transferred to polyvinylidene difluoride (PVDF, Merck Millipore, Billerica, MA, USA) membranes. The membranes were blocked with nonfat milk at room temperature for 1 h and incubated with primary antibodies against GSDMD, cleaved GSDMD and β-actin at 4 °C overnight. After washing, the membranes were incubated for 1 h with HRP-conjugated secondary antibodies at room temperature and visualized using electrochemiluminescence (ECL) western blotting substrate reagent (Perkin-Elmer, Waltham, MA, USA) following the manufacturer’s instructions. The protein bands were analyzed using ImageJ software (version 1.8.0) and the expression of the proteins normalized to the loading control β-actin.

### Immunohistochemistry analysis

Paraffin-embedded renal tissue sections were incubated with 3% hydrogen peroxide for 30 min and blocked with 5% goat serum for 1 h, followed by being stained with a rabbit anti-mouse NLRP3, caspase-1, cleaved caspase-1, or GSDMD antibody overnight at 4 °C. Thereafter, the sections were washed with PBS and incubated with an HRP-conjugated secondary antibody at room temperature for 1 h. Subsequently, these sections were stained with the DAB working solution and counterstained with Hematoxylin. All images were acquired by an upright microscope (Carl Zeiss, Jena, Germany).

### Immunofluorescence staining

Immunofluorescence staining was performed to measure the expression of NLRP3, cleaved caspase-1, and caspase-1 in THP-1 cells. Firstly, the cells were blocked with 5% goat serum and incubated with a rabbit monoclonal antibody against NLRP3, caspase-1, or cleaved caspase-1 overnight at 4 °C. Then the sections were washed with PBS three times and sequentially stained with TRITC-conjugated anti-rabbit IgG at room temperature for 2 h. Cell nuclei were stained with 4′, 6-diamidino-2-phenylindole.

To assess the expression of GSDMD in macrophages, double immunofluorescence staining was performed. Firstly, frozen sections of kidney tissues were blocked with 5% goat serum and incubated with a rabbit monoclonal antibody against GSDMD as well as a rat monoclonal antibody against F4/80 overnight at 4 °C. Then the sections were washed with PBS three times and sequentially stained with TRITC-conjugated anti-rabbit IgG and FITC-conjugated anti-rat IgG at room temperature for 2 h. Cell nuclei were stained with 4′, 6-diamidino-2-phenylindole. Images were taken using a fluorescence microscope.

### Statistical analysis

All data in this experiment were analyzed by SPSS software (version 23.0) and the figures drawn with Graphpad Prism (version 7.0). Independent-samples T-test, one-way ANOVA, or the non-parametric Wilcoxon rank-sum test were used according to data distribution. Data are displayed as the mean ± SD. *P* values < 0.05 was defined as statistically significant.

## Supplementary information

Original western blots

Original western blots

Original western blots

## Data Availability

The datasets generated during and/or analyzed during the current study are available from the corresponding author on reasonable request.
