# Supplementary figures and images for: Disulfiram alleviates pristane-induced lupus via inhibiting GSDMD-mediated pyroptosis
Source: Cell Death Discov. 2022 Sep 3;8:379. doi: 10.1038/s41420-022-01167-2 (PMC9440918; doi:10.1038/s41420-022-01167-2)

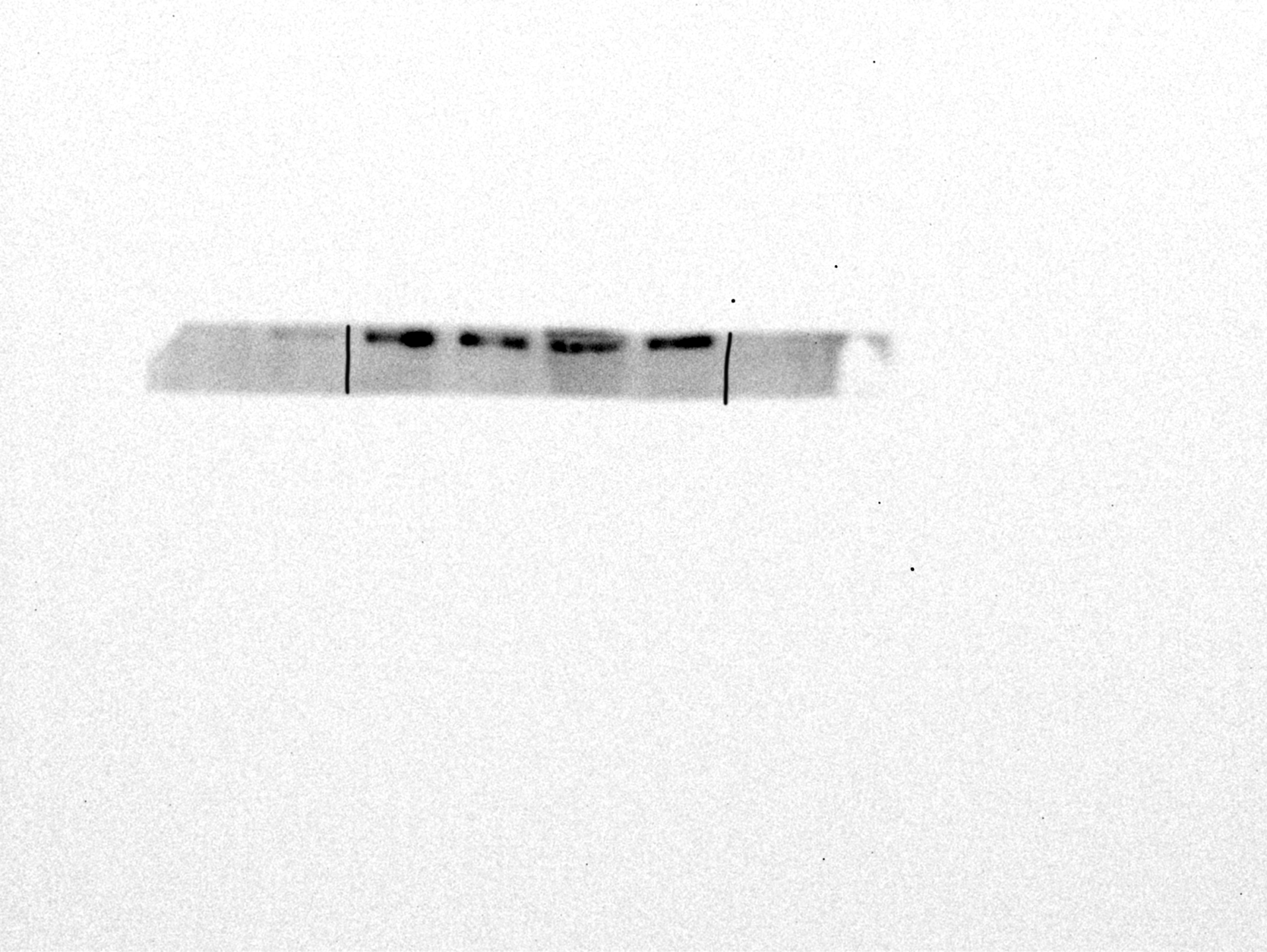

Supplement: Supplementary file 1 — Original western blots [file 41420_2022_1167_MOESM1_ESM.png]

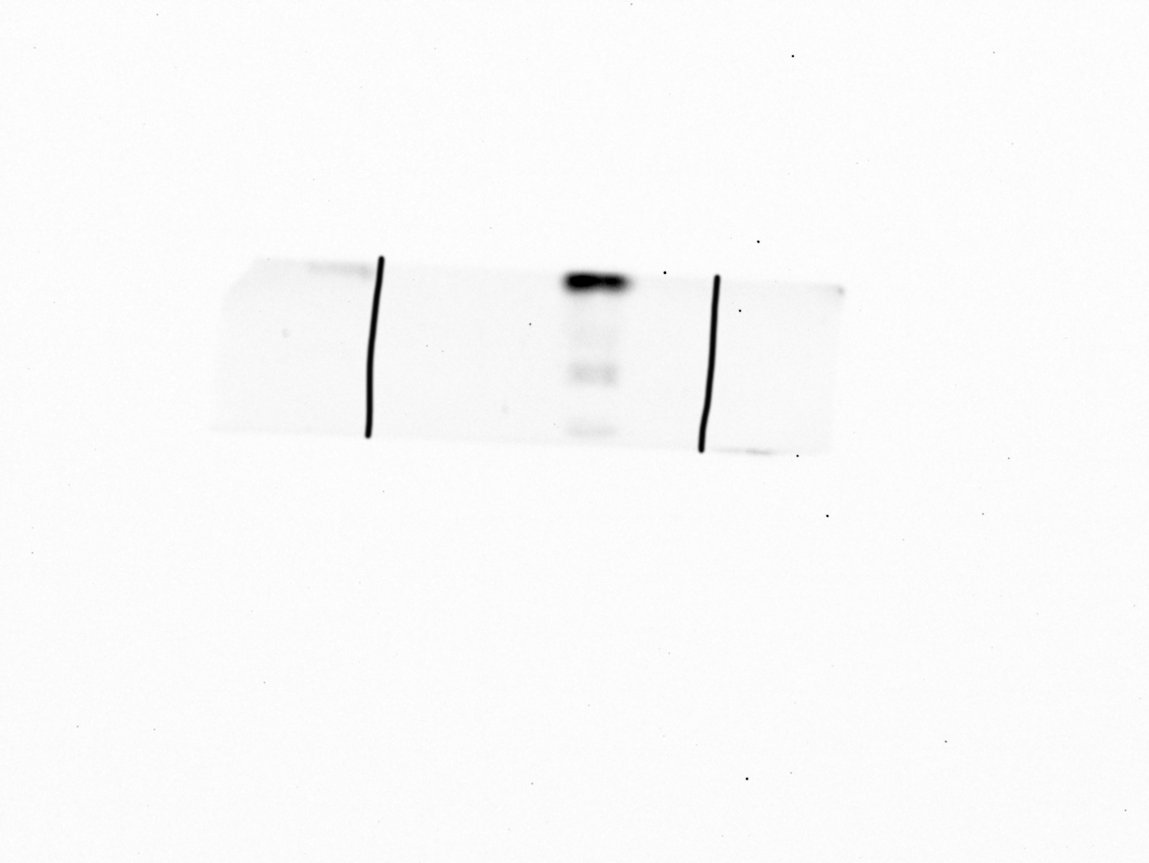

Supplement: Supplementary file 2 — Original western blots [file 41420_2022_1167_MOESM2_ESM.png]

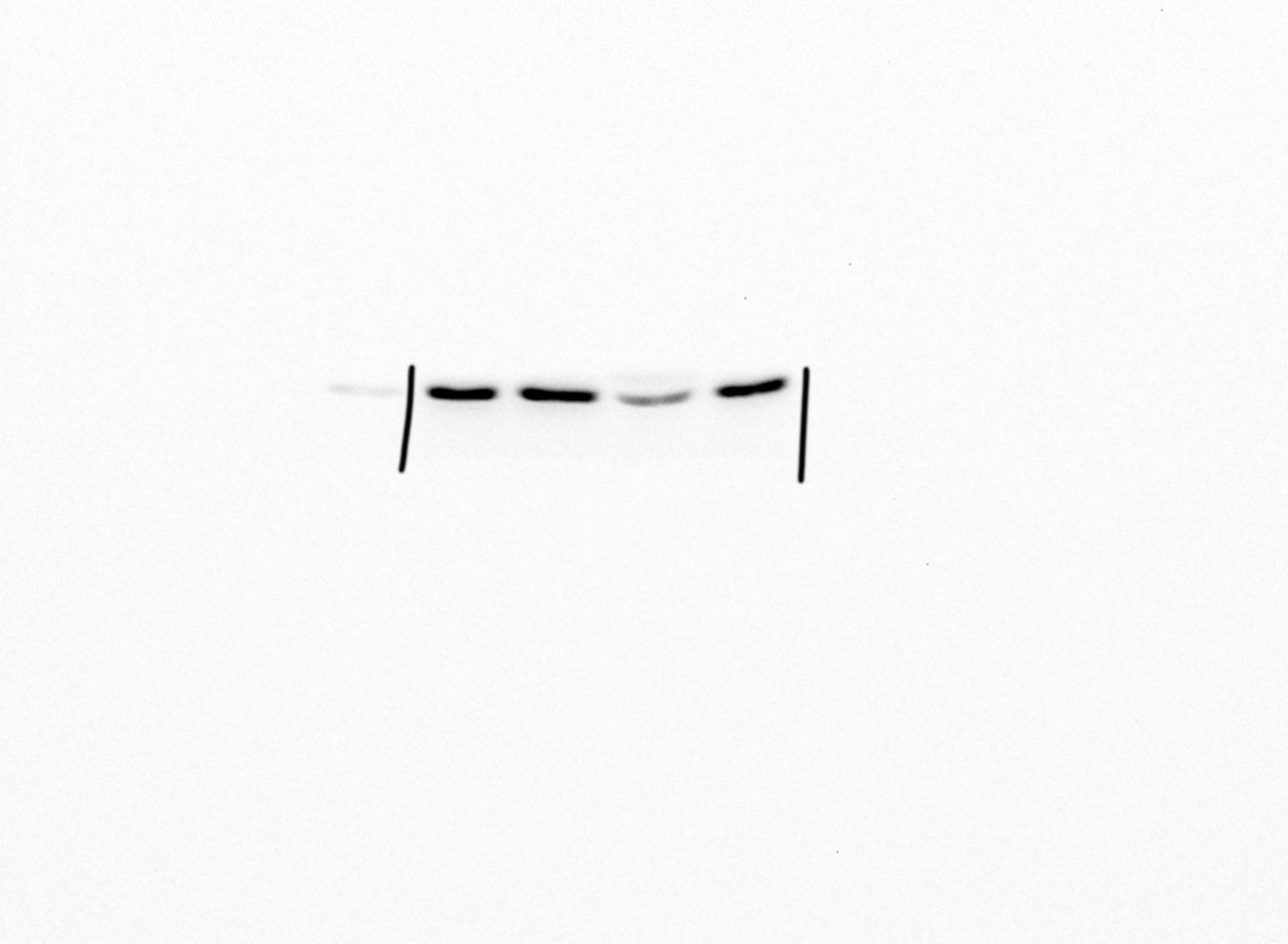

Supplement: Supplementary file 3 — Original western blots [file 41420_2022_1167_MOESM3_ESM.png]
